# Acute-onset Flu Encephalopathy in an Adolescent Female

**DOI:** 10.7759/cureus.4485

**Published:** 2019-04-17

**Authors:** Peter K Ballard, Sean M O'Hara, Erica M Simon

**Affiliations:** 1 Emergency Department, Brooke Army Medical Center, San Antonio, USA

**Keywords:** encephalopathy, emergency medicine, altered mental status, pediatrics, pediatric emergency medicine

## Abstract

Flu encephalopathy is a rare and poorly understood complication of the influenza virus. In children, it presents most commonly in the 6-18 months age range and most often in the first 26 hours of flu symptoms. Here, we present a case of a 13-year old black female who presented with acute-onset encephalopathy two weeks into flu symptoms. As we begin this flu season, this case serves as a reminder that flu encephalopathy should be on the differential for acute-onset altered mental status.

## Introduction

Flu encephalopathy is a rare and poorly understood complication of the influenza virus. In children, it presents most commonly in the 6-18 months age range and most often in the first 26 hours of flu symptoms. Typical symptoms include altered mental status (AMS), convulsions, upper respiratory symptoms, and vomiting. It carries a 30% mortality rate while a third of the remaining patients experience permanent neurologic sequela [1]. The syndrome has been best described in Asian countries and less often in Caucasian children [2]. The incidence of the disease is low (estimated at 0.21 per million cases), but it has globally increased since the hemagglutinin one neuraminidase one (H1N1) epidemic [3]. Though the pathophysiology is still not well-understood, it has been ascribed to serum pro-inflammatory cytokine burden [4-5].

## Case presentation

A 13-year-old female without a significant previous medical history presented unresponsive to a level I trauma center. The patient was fully vaccinated with the exception of the seasonal flu vaccine. Per the parent’s report, the patient had experienced cough and cold symptoms for two weeks. Thirty minutes following the patient’s departure to bed, she was heard screaming. Upon responding to the patient’s cry, her parents discovered her minimally responsive and having vomited. With significant assistance, she was able to walk to their car. Upon arrival at the emergency department, the patient was completely non-responsive. Initial vitals were a temperature of 35.2 degrees Celsius, pulse 70, blood pressure 117/65, respiratory rate 12, and saturation 100% on room air. Upon examination, the patient was Glasgow Coma Scale (GCS) three, breathing spontaneously and with a bounding pulse. Pinpoint pupils and a disconjugate gaze were noted. Intravenous naloxone 0.4 milligrams (mg) was administered without a change in mental status, and a subsequent 1 mg dose resulted in no further improvement. Non-contrasted computed tomography (CT) was read as suggestive of a small perimesencephalic bleed, but nothing that should be causing her symptoms.

Laboratory studies revealed leukocytosis (white blood cell count of 15.5 x 10^3/microliter). In conjunction with the patient’s hypothermia, antibiotics were initiated empirically (systemic inflammatory response syndrome present; sepsis presumed with the most likely etiology being meningitis). An acetaminophen level of 138 micrograms/millilitre was identified. Acetylcysteine was initiated to address a possible chronic acetaminophen toxicity (the assumption being that she had been chronically treating her symptoms with acetaminophen). A lumbar puncture (LP) was obtained, and a meningitis encephalitis polymerase chain reaction (PCR) study ordered. Initial cerebral spinal fluid results (glucose 85/100milliliter, protein 31/100milliliter, and cell count Ω of three red blood cells and one polymorphonuclear neutrophil) were all within normal limits. The decision was made to intubate the patient as her mental status was not improving and she had begun to vomit again. The patient was then admitted to the pediatric intensive care unit with influenza A and B PCR pending. Shortly after her arrival at the pediatric intensive care unit (PICU), the influenza A PCR resulted positive. The patient’s inpatient course included a magnetic resonance angiogram (MRA) of the brain and electroencephalogram (EEG). The MRA was unremarkable. This study, in combination with LP results non-suggestive of a subarachnoid hemorrhage, suggested that the earlier concern for a perimesencephalic bleed was a false positive. Her EEG revealed “intermittent generalized slowing consistent with toxic metabolic encephalopathy.” Flu encephalopathy was determined to be the etiology of the patient’s altered mental status. Incidentally, further laboratory studies showed that the patient was infected with the H3 influenza A variant which was previously associated with an increased incidence of flu encephalopathy in Japan in the 1990s [5].

After 72 hours in the PICU on intravenous peramivir, the patient’s mental status improved significantly; she was extubated, and in the days following, had a complete neurologic recovery. Her normal magnetic resonance imaging (MRI) and her subsequent full recovery are consistent with the prior finding, in adult patients, that a normal MRI is of prognostic value [3].

## Discussion

This patient presented with acute onset AMS that rapidly progressed to a comatose state. The differential initially included trauma, toxicologic, and intracranial hemorrhage with specific supportive therapies initiated as information became available.

A diagnosis of exclusion, flu encephalopathy, was ultimately identified. Influenza is difficult to detect on cerebral spinal fluid PCRs and is only positive in approximately 16% of patients [3]. After excluding immediate life threats with a typical AMS evaluation (including CT/LP, toxicologic studies, evaluation for trauma), additional diagnostic testing for flu encephalopathy typically includes an MRI/MRA and EEG. The MRI can be normal, but the presence of lesions in the cerebellum, brainstem, or thalamus have previously been identified in other patients. The presence of these lesions is a poor prognostic factor, as patients with an abnormal MRI have a full recovery 50% of the time, as opposed to 78% of patients having a full recovery if their MRI is normal [6]. The EEG typically displays global slowing, which is non-diagnostic, but supportive of a general encephalopathic process. Had the influenza PCR panel been sent earlier, or flu encephalopathy been on our initial differential, antivirals could have been started in the emergency department.

## Conclusions

Thankfully, this patient made a full recovery without sequelae. She serves as a reminder, as we approach flu season, that flu encephalopathy should be on the differential for acute-onset altered mental status.

## References

[1] Surtees R (2006). Influenza virus associated encephalopathy. Arch Dis Child.

[2] Kirton A, Busche K, Ross C, Wirrell Wirrell, E E (2005). Acute necrotizing encephalopathy in caucasian children: two cases and review of the literature. J Child Neurol.

[3] Meijer WJ, Linn FHH, Wensing AMJ (2016). Acute influenza virus-associated encephalitis and encephalopathy in adults: a challenging diagnosis. JMM Case Rep.

[4] Lee N, Wong CK, Chan PKS (2010). Acute encephalopathy associated with influenza A infection in adults. Emerg Infect Dis.

[5] Morishima T (2004). Influenza-associated encephalopathy. Rinsho Shinkeigaku.

[6] Studahl M (2003). Influenza virus and CNS manifestations. J Clin Virol.

